# Brucellosis in a refugee who migrated from Syria to Germany and lessons learnt, 2016

**DOI:** 10.2807/1560-7917.ES.2016.21.31.30311

**Published:** 2016-08-04

**Authors:** Roland Grunow, Daniela Jacob, Silke Klee, Dietmar Schlembach, Sabine Jackowski-Dohrmann, Vera Loenning-Baucke, Bettina Eberspächer, Sonja Swidsinski

**Affiliations:** 1 Robert Koch Institute, Berlin, Germany; 2 Vivantes Klinikum Neukölln, Geburtsmedizin, Berlin, Germany; 3 Vivantes Klinikum Neukölln, Kinder und Jugendmedizin, Berlin, Germany; 4 Charité Hospital, Campus Charité Mitte, Laboratory for Molecular Genetics, Polymicrobial Infections and Bacterial Biofilms, Berlin, Germany; 5 Labor Berlin, Berlin, Germany

**Keywords:** Brucella, brucellosis, clinic, emerging or re-emerging diseases, refugee, biosafety

## Abstract

A teenage woman migrating from Syria arrived in May 2015 in Germany. She gave birth to a healthy child in early 2016, but became febrile shortly after delivery. Blood cultures revealed *Brucella melitensis.* In retrospect, she reported contact with sheep in Syria and recurrent pain in the hip joints over about five months before diagnosis of brucellosis. We discuss consequences for adequate treatment of mother and child as well as for clinical and laboratory management.

As brucellosis is rare in many European countries and because there is even less experience with brucellosis in association with pregnancy, we here publish a case of a young pregnant woman who had migrated from Syria to Germany and was diagnosed with brucellosis directly after delivery of her child, and illustrate some lessons learnt. 

## Case report

A teenage female refugee from Syria delivered a full-term baby (birthweight 3,185 g) with vacuum extraction in Germany. Because of intrapartum asphyxia, mother and child were hospitalised in a special care unit. The child recovered without further complications. The mother developed a fever of 39 °C on day 1 after delivery. Investigation of the mother’s blood revealed normal ranges of leukocytes, a slightly decreased haemoglobin level and an elevated concentration of C-reactive protein (CRP) at 38.0 mg/mL (normal < 5.0 mg/L). She was treated for three days with intravenous ampicillin sulbactam 3 g three times daily and metronidazole 500 mg twice daily. The patient was in good clinical condition and discharged from the hospital on day 4 with oral sultamicillin 375 mg and metronidazole 400 mg twice daily.

**Figure 1:**
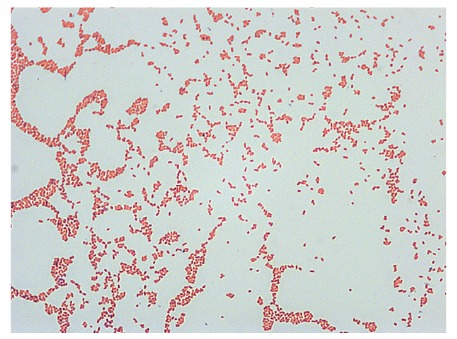
Gram staining of bacterial culture showing faintly stained small Gram-negative coccobacilli corresponding to *Brucella* spp., Germany, 2016

**Figure 2:**
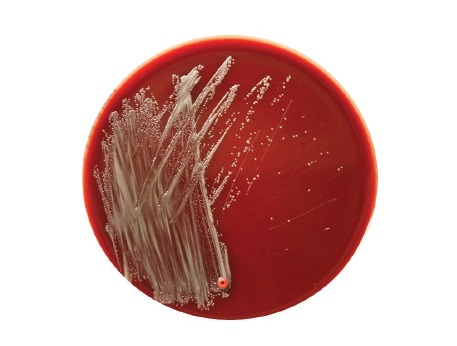
Bacteria isolated from blood culture forming small non-haemolytic colonies on Columbia blood agar after 48 hours incubation at 36 °C air with 5% CO_2_ enrichment, Germany, 2016

Two sets of blood cultures (BD Bactec Fx blood culture system) were taken during pyrexia. One anaerobic culture revealed a positive signal after 47 h incubation that was identified as *Fusobacterium nucleatum*. The aerobic blood culture revealed growth after 117 h and Gram staining showed faintly stained small Gram-negative coccobacilli (Figure 1). Small non-haemolytic colonies appeared on Columbia blood agar after 48 hours incubation at 36 °C in air with 5% CO_2_ enrichment (Figure 2) and were oxidase-positive. Using matrix-assisted laser desorption ionisation time of flight (MALDI-TOF) mass spectrometry (MS) (MALDI-TOF MS, Vitek MS Plus System bioMérieux, research use only (RUO) SARAMIS database), *Brucella* spp. was identified and confirmed as *Brucella melitensis* one day later when tested by real-time PCR in a reference laboratory using an accredited in-house assay.

Because of the results of the aerobic blood culture, the mother and child were immediately contacted and examined on day 11 after delivery. The mother reported that she was a sheepherder in Syria before arriving in Germany nine months previously and that she had suffered from recurrent hip pain for about five months before diagnosis of brucellosis. The serum sample was tested in parallel with two ELISAs revealing a high titre of 1:16,000 (normal: < 1:500; Virion-Serion GmbH, Würzburg, Germany) of *Brucella*-specific antibodies, with IgG of 29.1 U and IgM of 24.3 U (cut-off: 10 U; Novagnost Brucella IgG and IgM ELISA, Siemens Healthcare Diagnostics, Germany). Samples of mother’s milk were not available for laboratory investigation. The patient was advised to start antibiotic therapy with doxycycline 100 mg twice daily and rifampicin 900 mg once daily for 12 weeks and to stop breastfeeding.

The examination of the baby by the paediatrician revealed no relevant clinical or laboratory abnormalities. The blood culture set up day 11 after birth remained negative, the serum sample was positive only for Brucella-specific IgG antibodies (24.5 U).

In subsequent visits (four and seven weeks after delivery), mother and child had no relevant clinical or laboratory abnormalities. The antibiotics were well tolerated by the mother. Blood cultures from the mother drawn in week 7 after delivery remained negative; the IgM titre was slightly decreased to 18 U.

After the identification of *Brucella* spp., two persons from the delivery room were monitored and had serological follow-up. All serological tests at their first visit and three months later were negative.

## Background

Brucellosis is a zoonosis that occurs worldwide, causing great economic losses and a high number of human infections in endemic regions, such as the Mediterranean, the Middle East including Syria, Iraq, Iran and Saudi Arabia, as well as Africa, Asia and Middle and South America. It is estimated that more than 500,000 people are suffering from brucellosis annually [1]. In contrast, only 22 to 47 cases annually were reported in Germany between 2010 and 2015. Most of these cases were associated with travel to endemic countries surrounding the Mediterranean Sea (Italy, Spain, Turkey). Between January 2015 and June 2016, 60 cases of brucellosis in Germany were reported to the Robert Koch Institute, 14 from the Middle East and North African countries, including four cases from Turkey. This seems to point towards a tendency of increased importations from these regions. Imported brucellosis plays an emergent role in Europe and globally [2,3].

*B. melitensis* (goats and sheep) is the most important species for human brucellosis, but other species like *B. abortus* (cattle), *B. suis* biovar 1 (swine) and *B. canis* (dogs) can also be associated with human cases. The small Gram-negative coccobacilli are most commonly transmitted to humans through contaminated food, e.g. unpasteurised milk, or by direct contact with infected animals [4,5]. They grow intracellularly, e.g. in mononuclear phagocytic cells, cause unspecific clinical manifestations especially in the acute stage, including typically undulant fever, which may last for years if not properly treated. The bacteria can be disseminated to various organs such as liver, spleen, bones and joints, genitourinary tract, skin, lung, heart and central nervous system. Therefore, human brucellosis has a broad variability of clinical signs and can mimic other infectious and non-infectious diseases. The infection is often diagnosed only when focal complications occur. Symptoms in children and teenagers with brucellosis are mostly fever, joint pain and hepatomegaly [6,7]. Intrauterine infections and transmission of the pathogen through breastfeeding has been reported, transmission of antibodies from mother to newborn is possible [7].

The laboratory diagnosis is mainly based on classical microbiological methods (but isolation could be cumbersome and is not always successful), on MALDI-TOF MS identification of colonies and on PCR that allows the discrimination of *Brucella* species [8-13]. In addition, multilocus variable-number tandem repeat analysis and other methods like whole genome sequencing, next generation sequencing and single nucleotide polymorphism analysis allow the generation of molecular epidemiology data that can be helpful in the identification of outbreak-related strains and the geographical relation between isolates [14,15]. The detection of IgG and IgM antibodies against *Brucella* antigens in serum is a reliable tool for diagnosing human brucellosis and classifying the stage of disease [16,17]. IgM anti-*Brucella* antibodies indicate acute infection, and a titre increase in paired serum samples indicates an acute or reactivated infection.

## Discussion

The accidental cultivation of *Brucella melitensis* in blood cultures drawn during post-partum fever demonstrates the complexity of this diagnosis. The anamnestic, clinical and laboratory data of our patient were consistent with a protracted or chronic course of brucellosis activated during pregnancy and/or delivery. However, the exact point in time and place of infection of our patient cannot be determined. As she was working as sheepherder in her home country before coming to Germany, she may have been infected in Syria, but infection along the route of migration is also possible. Prolonged antimicrobial therapy is imperative for achieving cure; monotherapy is associated with a high rate of relapse. In this case, the 12-week regimen based on oral doxycycline 100 mg twice a day in combination with rifampicin 900 mg/day in a single dose was preferred because of its oral application and fewer adverse effects compared to the combination of doxycycline with aminoglycosides (such as streptomycin or gentamicin).

Because of possible transmission either transplacentally or through breastfeeding (during the 11 days until diagnosis), the baby was examined. Antibacterial treatment was not started for the baby because there were no relevant clinical or laboratory abnormalities. Regular examinations, at least every three months over a period of one year, were advised.

Although person-to-person transmission of brucellosis is rare, transmission may occur in contact with contaminated blood and by aerosol-producing diagnostic procedures [18]. For patients suffering from human brucellosis during hospitalisation, standard precautions and contact precautions for those with draining wounds are required. In the case described here, mother and newborn were in a rooming-in unit and under contact precautions because of hygiene regulations for refugees.

Handling of cultures with human pathogenic *Brucella* spp. requires biosafety level 3 (BSL3) conditions, i.e. consequent work in a class II biologic safety cabinet. However, for primary diagnostic samples, accidental cultivation of *Brucella* spp. in a routine (BSL2) laboratory cannot be avoided. This entails working without safety cabinets for reading agar plates, performing phenotypical tests, etc. It was estimated that brucellosis represents the most common laboratory-acquired infection in clinical routine laboratories [19-21], and an enhanced safety policy are necessary to prevent laboratory acquisition of *Brucella* as well as regular training of the laboratory staff to be aware of how to handle and cultivate highly pathogenic bacteria such as *Brucella* spp. In our case, technicians had regularly been trained to recognise the combination of small faintly stained Gram-negative coccobacilli (‘fine sand’), slow-growing small colonies on Columbia blood agar and the requirement of aerobic conditions with CO_2_ enrichment, and were aware of the necessary biosafety measures. When *Brucella* spp. was suspected during reading of the agar plates, handling outside a safety cabinet was immediately stopped. All following steps for identification were performed under a class II biosafety cabinet with adequate precautions; as the risk of infection of laboratory personnel was high, we consider that these measures were effective in preventing an infection.

## Lessons learnt 

The clinical microbiological laboratory plays a key role in the diagnosis and management of human brucellosis. It should provide a rapid and exact identification of every Gram-negative rod that is cultivated from blood culture or surgical material to exclude *Brucella* spp. or other highly pathogenic bacteria. Currently, the most suitable tool for identification of bacteria is MALDI-TOF MS because it provides rapid, accurate, sensitive and cost-effective identification of human pathogenic bacteria. Attention should be paid using commercially available MALDI-TOF MS systems including standard diagnostic databases to identify *Brucella* spp. and other highly pathogenic bacteria. The related MALDI-TOF MS databases do not usually support validated laboratory diagnoses of highly pathogenic bacteria, which should be improved in the future. In addition, when highly pathogenic bacteria such as *Brucella* spp. are suspected or the results are ambiguous, material should be sent to a reference laboratory for further confirmation and identification at species level. When sending live material, the international dangerous goods regulations for air transportation (International Air Transportation Association (IATA)) [22] and ground transportation (Accord européen relatif au transport international des marchandises Dangereuses par Route (ADR)) [23] need to be considered. Alternatively, for PCR and genome analyses, sending bacterial DNA can be sufficient. Genetic material prepared by an evaluated protocol including safe inactivation of the pathogen can be sent without considering specific shipment regulations.

We can assume that physicians in non-endemic European areas have a poor awareness of imported brucellosis in patients arriving from regions with endemic brucellosis, exemplified in our case by people who have migrated from regions in the Middle East. Although our patient had been in Germany for nine months and in ambulatory care during pregnancy, questions on travel history, animal contacts and consumption of raw animal products or duration of symptoms were not asked [4,24]. A connection between hip pain and brucellosis was not made.
